# Silacyclohexanes, Sila(hetero)cyclohexanes and Related Compounds: Structure and Conformational Analysis

**DOI:** 10.3390/molecules25071624

**Published:** 2020-04-01

**Authors:** Bagrat A. Shainyan

**Affiliations:** A. E. Favorsky Irkutsk Institute of Chemistry, Siberian Branch of Russian Academy of Science, 1 Favorsky Street, Irkutsk 664033, Russia; bagrat@irioch.irk.ru

**Keywords:** conformational analysis, structure, silicon-containing heterocycles, GED, LT NMR, theoretical calculations

## Abstract

Conformational analysis of Si-mono- and Si,Si-disubstituted silacyclohexanes as well as their analogues with a heteroatom(s) in the ring is reviewed with the focus on the recent results. Experimental measurements in the gas phase (gas electron diffraction, GED) and low temperature NMR spectroscopy (LT NMR) on ^1^H, ^13^C and ^29^Si nuclei are described along with theoretical calculations at the DFT and MP2 levels of theory. Structural and conformational specific features are shown to be principally different from those of the carbon predecessors—the corresponding cyclohexanes, oxanes, thianes and piperidines. The role of various effects (steric, hyperconjugation, stereoelectronic, electrostatic) is demonstrated.

## 1. Introduction

Conformational analysis of cyclohexanes is an inalienable part of organic chemistry that is familiar to all organic chemists, even to those whose interests lie outside this specific area. In going from cyclohexanes to their N-, O-, or S-heteroanalogues, no dramatic changes occur in the structure or conformational preferences of the substituents, which may give an illusion that this is true for all other heterocyclohexanes. However, this is not always so, as it became clear in the last 15 years, when a large number of silacyclohexanes with different groups at silicon, as well as having one or more other heteroatoms in the six-membered ring, such as N, O, S, have been synthesized. The only review on the topic was that of 2004 [1] reporting a few compounds known at that time: silacyclohexane, 1,4-disila- and 1,3,5-trisilacylohexanes and their Si-alkyl derivatives. Since then only two reports published by the present author and reviewing specific questions appeared [2,3]. The nowadays state of the problem is unique in that, on the one hand, it still allows discussing all main findings in the field in one review, and, on the other hand, making solid conclusions based on ample experimental evidences. The studies using various experimental techniques, such as GED, LT NMR, Raman and IR spectroscopy and quantum chemical calculations showed a remarkable difference in the structure and conformational behavior between sila(hetero)cyclohexanes and cyclohexanes, piperidines, oxanes, or thianes. The present review summarizes the results of experimental and theoretical conformational studies on the title compounds in different aggregate states, and is focused on recent works in this field. All these issues will be addressed in the proposed review providing the reader with the answers to the raised questions. The list of references contains 93 items including those of 2019 year, most of them referring to the last decade.

## 2. General Features of Sila(hetero)cyclohexanes

The three main issues in conformational analysis of cyclohexanes and all their derivatives are the ring conformation, the ratio of the conformers, and the barrier to their interconversion. As will be shown below, these three characteristics of sila(hetero)cyclohexanes drastically differ from those of cyclohexanes, piperidines, oxanes, or thianes.

**Figure 1:**
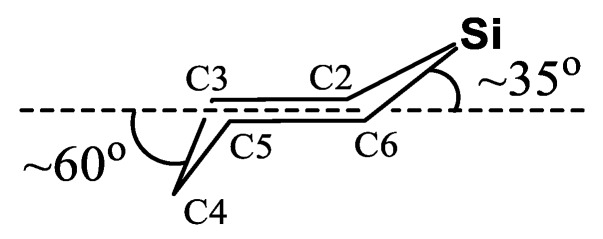
Lower degree of folding at silicon in the six-membered ring.

As to the ring conformation, an important difference is a smaller angle of folding between the C2C3C5C6 plane of the ring and the C2SiC6 plane with respect to the C3C4C5 plane as depicted in Figure 1. The same is true for the nitrogen, oxygen or sulfur-containing heterocycles, in which atom C4 is replaced by the corresponding heteroatom [4].

The ratio of the conformers is also dramatically different. It is determined by the relative conformational energies, ***A***, defined for the *ax* ⇆ *eq* equilibrium as ***A*** = −Δ*G*° = *G*_ax_ − *G*_eq_. For cyclohexanes, piperidines, oxanes, or thianes the ***A*** values are always positive. For substituents at silicon they are much smaller and even may become negative. Most striking examples are practically equal ***A*** values for Me and Ph groups at silicon (0.23 [5] and 0.25 kcal/mol [6]), whereas in the cyclohexane series they are strongly different (1.76 and 2.87 kcal/mol [7]), and the value of ***A*** for very bulky but, at the same time, highly electronegative substituent CF_3_, which is positive and very large when it is attached to carbon (2.50 kcal/mol [8]) but becomes negative for CF_3_ at silicon falling in the range from −0.2 to +0.5 kcal/mol [9]. The ring inversion barriers in silacyclohexanes and their analogues are much lower than those in the carbon predecessors. Normally, they are as low as 4.5–5.5 kcal/mol, in comparison to 10–14 kcal/mol in cyclohexanes, piperidines, oxanes, or thianes. Such small barriers can be measured by NMR only by using special NMR probes working at very low temperatures, down to the interval from −180 to −190 °C, and solvents, which are not frozen at these temperatures (mixtures of freons). Such low barriers are due to the flattened structure of sila(hetero)cyclohexanes (Figure 1), which is closer to the transition state structurally and, according to the Hammond postulate, energetically, and, hence, needs less energy to reach it.

Other differences of sila(hetero)cyclohexanes, like additivity of conformational effects versus nonadditivity in cyclohexanes, or opposite conformational preferences in gas and solution, having no precedents in classical conformational analysis, will be discussed below.

## 3. Silacyclohexanes

**Figure 2:**
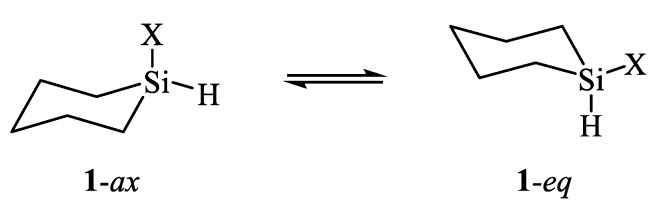
Conformational equilibrium of monosubstituted silacyclohexanes.

The conformational equilibrium of monosubstituted 1-X-silacyclohexanes **1** in Figure 2 can be shifted either to the axial or to the equatorial conformer, depending on the nature of substituent X. Therefore, the conformational energy of X, ***A***(X)_Si_ = −Δ*G* = −(*G*_eq_ − *G*_ax_) = R*T* log([*eq*]/[*ax*]), can be either positive or negative, in contrast to the corresponding cyclohexanes, in which ***A***(X)_C_ is always positive, that is, the substituent always prefers the equatorial position. The available ***A*** values for both series are summarized in Table 1.

The following conclusions can be made from analysis of the data in Table 1. First, a drastic decrease of all ***A*** values in going from cyclohexanes to silacyclohexanes implies a minor, subordinate role of steric effects in silacyclohexanes. Second, inversion of the sign (X = halogen) or sharp decrease (X = CF_3_) of the ***A*** values of these electronegative groups is indicative of the determining role of electrostatic effects in the latter series. This is owing to the presence of strongly electropositive silicon atom in the molecule and is clearly manifested not only in the position of the equilibrium between the conformers but also in the lengths of the Si-C bonds. Thus, the Si-CF_3_ bond in trifluoromethylsilacyclohexane is elongated by ~0.08 Å relative to the Si-CH_2_ because of repulsion of the positively charged Si and CF_3_ carbon atoms.

The same is true for the Si-CF_3_ (1.934 Å) and Si–Me bond in 1-methyl-1-silacyclohexane (1.862 Å). The relative importance of steric, hyperconjugation and electrostatic effects was evaluated and discussed in detail in the author’s review [3]. A minor role of steric effects not veiled by the effect of strongly electronegative groups (halogen, CF_3_) can be followed on the example of X = SiH_3_. Low ***A*** in silylcyclohexane (0.33) is, evidently, due to the long C–Si bond (~1.85 Å). Further lengthening in 1-silylsilacyclohexane [e.g., in (*t*-BuSi)_2_ the length of the Si–Si bond is 2.69 Å) makes the steric effect negligible (***A*** = 0.05 kcal/mol). The only substituent, for which steric effect could be significant in silacyclohexanes is *t*-butyl group, but until recently the only available compound of this type was the simplest representative, 1-*t*-butylsilacyclohexane. Very recently we have synthesized 1-*t*-butyl-1-phenylsilacyclohexane and investigated its conformational equilibrium (*vide infra*).

**Figure 3:**
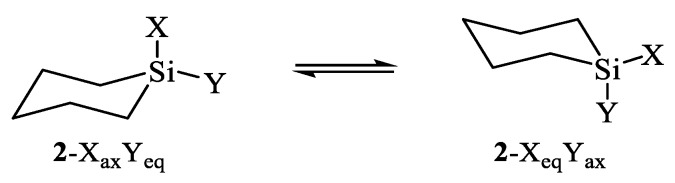
Conformational equilibrium of geminally 1,1-disubstituted silacyclohexanes.

Introduction of a second substituent to silicon rises two interrelated questions in conformational analysis of geminally 1,1-disubstituted silacyclohexanes **2**: (i) qualitative applicability of individual ***A*** values of substituents X and Y for prediction of the position of conformational equilibrium in Figure 3, and (ii) quantitative prediction of the conformer ratio on the basis of monosubstituted compounds (additivity or nonadditivity of conformational energies).

It makes sense to compare the conformational behavior of geminally substituted cyclohexanes and silacyclohexanes. As is clearly seen from Table 2, there is neither quantitative nor even qualitative agreement between the conformational preferences in the mono and disubstituted cyclohexanes. The mean deviation ΔΔ*G*° of the experimental free energy difference Δ*G*°_ax–eq_ from that calculated by the additive scheme Δ*G*°_add_ for the presented selection of cyclohexanes is >1 kcal/mol, which is very large for conformational equilibria. The pairs of substituents for which the experimental and calculated values of Δ*G*° have different sign, that is, the observed conformational preferences are opposite to those predicted from ***A*** values for monosubstituted compounds, deserve special mention. The most striking example is 1-methyl-1-phenylcyclohexane, which, in view of the much larger conformational energy of the Ph as compared to Me group must exist exclusively as a Me_ax_Ph_eq_ conformer.

However, as was found experimentally, the equilibrium of 1-methyl-1-phenylcyclohexane is substantially shifted to the Me_eq_Ph_ax_ conformer (72:28, Δ*G*°_ax–eq_ = 0.32, Table 2) [26]. The reasons of such behavior have been clarified using high level quantum chemical calculations [20]. In the Me_ax_Ph_eq_ conformer, both Ph-‘orthogonal’ and Ph-‘horizontal’ rotamers are destabilized by repulsive interactions of *ortho*-protons with either the Me group or α-CH_eq_ protons, while in the Ph-‘horizontal’ rotamer of the Me_eq_Ph_ax_ conformer the latter destabilization is avoided. For the sake of fairness, it should be noted that there are examples of additivity of conformational effects in cyclohexanes [27,28], but in general, the conclusion about the absence of additivity of the ***A*** values in geminally disubstituted cyclohexanes made ten years ago [18] is undoubtedly true.

At that time it was too bold to say anything about additivity or nonadditivity in silacyclohexane series because there only available examples were Si(Me,F)- and Si(Me,CF_3_)-silacyclohexanes. Nevertheless, in the same work, Arnason et al. stated that *for silacyclohexanes the model works remarkably well for this limited selection of substituents* [18]. Since then, a good deal of studies devoted to conformational preferences in sila(hetero)cyclohexanes have seen the light, as summarized in Table 3. The analysis of Table 3 clearly shows that, if to exclude some specific cases with endocyclic N and O atoms in the ring (No. 10, 12 13, given in bold, which will be discussed below), averaging of other ΔΔ*G*° values results in the mean value of 0.1 kcal/mol for other 12 pairs of conformers, or more than one order of magnitude less than in Table 2 for cyclohexanes.

Therefore, the additivity model does work for silacyclohexanes without exceptions, and also for sila(hetero)cyclohexanes, unless the latter contain strongly electronegative atom or group in the ring. The exceptions deserve separate consideration, which allowed to reveal the influence of stereoelectronic and electrostatic effects in these specific cases.

**Figure 4:**
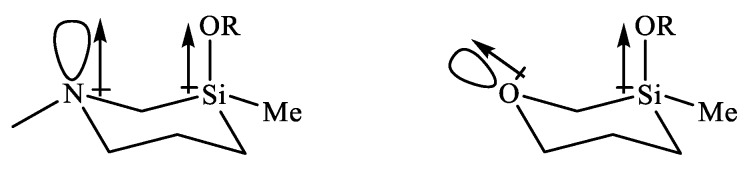
Orientation of the N and O lone pairs with respect to the O–Si dipole in 1,3-dimethyl-3-isopropoxy-1,3-azasilinane (**left**) and 3-isopropoxy-3-methyl-1,3-oxasilinane (**right**).

Large deviations from additivity in 1,3-dimethyl-3-isopropoxy-3-silapiperidine (No. 10, Table 3) were assigned to the repulsion of the two unidirectional axially oriented dipoles of the nitrogen lone pair and the highly polar Si-O bond (Figure 4, left), destabilizing the Me_eq_OR_ax_ conformer and making the Me_ax_OR_eq_ conformer predominant (the ratio was measured as 2:1) [31]. This effect is especially important in Si-alkoxy or Si-hydroxy-3-silapiperidines because of a large dipole moment of the Si-O bond and the higher basicity of nitrogen in α-silylamines relative to organic amines [36,37,38].

In 3-isopropoxy-3-methyl-1,3-oxasilinane (No. 12, Table 3), the Me_eq_OR_ax_:Me_ax_OR_eq_ ratio is 1:1, so, the deviation from additivity is smaller. The observed shift of the conformational equilibrium toward the Me_eq_OR_ax_ conformer is explained by orientation of the oxygen atom lone pair in the C-O-C plane [39], as shown in Figure 4, which reduces the repulsion between the two dipoles, as compared to that in the molecule of 1,3-dimethyl-3-isopropoxy-1,3-azasilinane.

**Figure 5:**
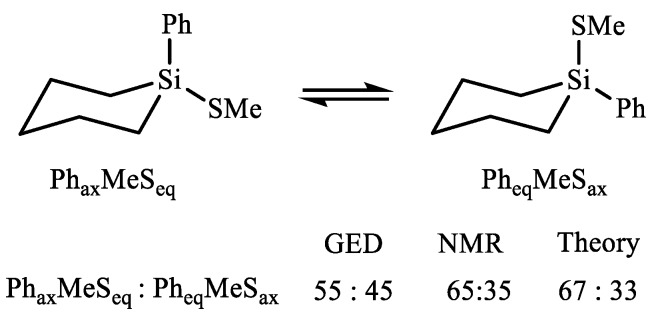
Ratio of 1-methylthio-1-phenyl-1-silacyclohexane conformers by different methods.

Very recently, we have synthesized 1-methylthio-1-phenyl-1-silacyclohexane and performed its conformational analysis using GED, LT NMR, and quantum chemical calculations [40]. It should be said that this was the first compound of this family that contained the exocyclic sulfur-containing group at silicon. It was prepared in 55% yield by the replacement of chlorine in 1-chloro-1-phenyl-1-silacyclohexane with MeSNa. The results are summarized in Figure 5.

**Figure 6:**
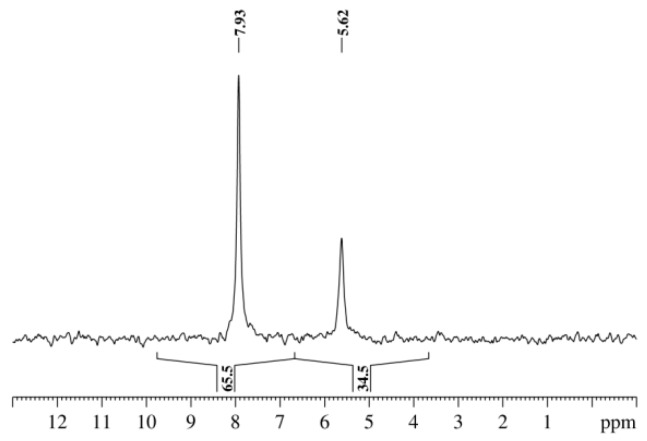
^29^Si-NMR spectrum of 1-methylthio-1-phenyl-1-silacyclohexane **1** at 98 K.

As follows from Figure 5, all methods clearly show the predominance of Ph_eq_ conformer, slightly larger in solution than in gas phase and in nice agreement with theoretical calculations. It must be said that LT NMR spectra were taken not only for ^1^H and ^13^C but also for ^29^Si (Figure 6) [40]. Until 2019, ^29^Si NMR was not used for conformational analysis of silacyclohexane or any other compounds. The first example was reported in our recent work at the same time (*vide infra*).

## 4. Thiasilacyclohexanes 

**Figure 7:**
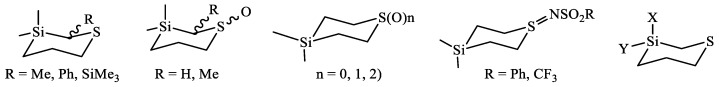
1,3- and 1,4-thiasilacyclohexanes and their S-functional derivatives.

The largest family of silaheterocyclohexanes is 3- and 4-silathianes including their S-functional derivatives (Figure 7).

Smaller conformational energies ***A*** are inherent not only to substituents at silicon, but, because of longer C−Si and C−S bonds, also to the groups attached to the endocyclic carbon atoms. For example, while the value of ***A***_Me_ is 1.76 kcal/mol (Table 1), the measured ratio of 2-Me_eq_:2-Me_ax_ conformers in the molecule of 2,3,3-trimethyl-3-silathiane is 60:40, that corresponds to Δ*G*^o^ = 0.35 kcal/mol, coinciding with the calculated value of 0.36 kcal/mol [41]. The analogues of the latter compound with 2-Me_3_Si [41] and 2-Ph substituents [42] exist as single 2-eq conformers due to the large conformational energy of the Ph (2.87 kcal/mol, Table 1) and SiMe_3_ group (experimental 2.5 kcal/mol, [43], theoretical 2.05 kcal/mol [44].

An interesting effect of the ring silicon atom was found when analyzing 3-silathiane S-oxides. The predominant existence of cyclic sulfoxides in the SO_ax_ conformation is well known [45]. Introduction of substituents at the 3-position to sulfur either in thiane or 3-silathiane destabilizes the SO_ax_ form, and both 3,3-dimethylthiane 1-oxide [46,47] and 3,3-dimethyl-3-silathiane S-oxide [46] exist in solution completely in the SO_eq_ form.

**Figure 8:**
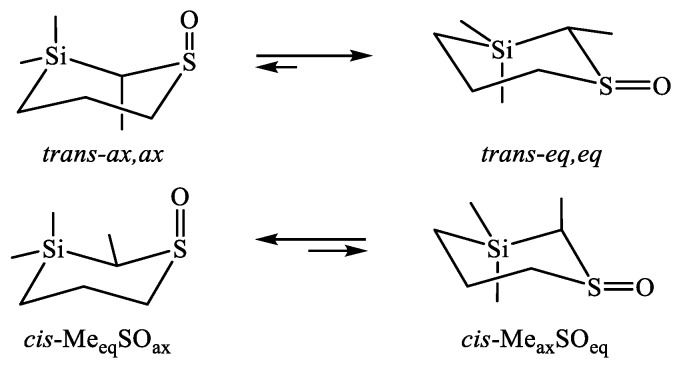
Conformational equilibria in the *trans* and *cis* isomers of 3-silathiane S-oxides.

The situation becomes more complicated when another substituent is introduced into the ring. Thus, the molecule of 2,3,3-trimethyl-3-silathiane S-oxide having two chiral centers (atoms C-2 and S), can exist as two diastereomers. Indeed, oxidation of 2,3,3-trimethyl-3-silathiane gives rise to the *cis*/*trans* diastereomeric mixture of the corresponding S-oxides in 1:2 ratio. The *trans*-isomer can adopt only the *eq-eq* form, but for the *cis*-isomer the Me_eq_SO_ax_ and Me_ax_SO_eq_ are in equilibrium (Figure 8) with the ratio Me_eq_SO_ax_:Me_ax_SO_eq_ equal to 1:1 at room temperature or 5:1 at −120 °C. This is consistent with the large ***A***_Me_ value (1.76 kcal/mol) and a small negative value of ***A***_SO_ (−0.18 kcal/mol). Although the presence of silicon in the ring lowers the ***A***_Me_ value and makes the ***A***_SO_ value positive, the Me_eq_SO_ax_ conformer is still preferable at low temperatures [48].

**Figure 9:**
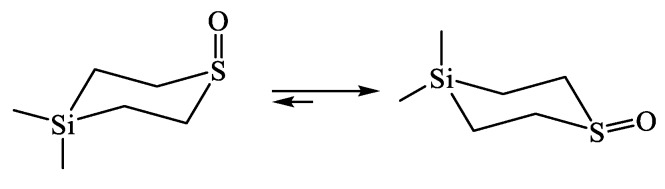
Predominance of the SO_eq_ conformer for 4-silathiane S-oxide.

In 4-silathiane S-oxides, the situation is different (Figure 9). The conformational equilibrium in solution is shifted to the equatorial conformer, the SO_eq_:SO_ax_ being 63:37, which is comparable with the 55:45 ratio found for thiane S-oxide [47].

Interestingly, theoretical DFT calculations showed the SO_ax_ conformer to be more stable by 0.93 kcal/mol [49], which seems to contradict the experiment. However, due to a larger dipole moment of SO_eq_ (5.41 vs. 4.21 D in SO_ax_) the use of the PCM in CHCl_3_ as the solvent led to the inversion of the relative stability and the SO_eq_ conformer was found to be 0.19 kcal/mol more stable [49]. This gives the ratio SO_eq_:SO_ax_ of 70:30 in excellent agreement with the experiment.

**Figure 10:**
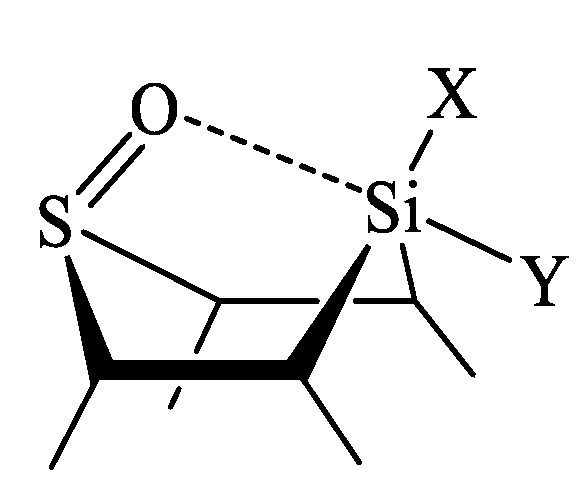
‘Scorpionoid’ *boat* structure of axial 4-silathiane S-oxides bearing halogens at silicon.

Theoretical studies of 4-silathiane S-oxides posed an interesting question about their molecular structure. Usually, sila(hetero)cyclohexanes adopt *chair* conformation. However, in the presence of one or two halogen atoms at silicon, the *boat* conformer may become a local minimum on the potential energy surface due to intramolecular S=O∙∙∙Si coordination, as shown in Figure 10.

For these structures, we coined the term ‘scorpionoids’, in which the silicon atom resembles the head and the sulfoxide oxygen atom the stinging tail. The relative stability of the *chair* and *boat* forms of 4-silathiane S-oxides with one or two halogens at silicon was analyzed in [50] (see also references cited therein). In the pentacoordinate motif around the silicon atom, the O···Si distance for various pairs of halogens (X, Y = H, F, Cl, Br) fall in the range of 2.05–2.15 Å, and is much less than the sum of the vdW radii of the two atoms (3.62 Å). Penta-coordination is also confirmed by the close to planar CCX equatorial arrangement around silicon, and by longer axial bond Si-Y than the equatorial bond Si-X in the bipyramidal silicon coordination knot [50]. We know only one example of the S=O···Si coordination in which the trigonal bipyramidal structure was proved experimentally [51].

4-Silathiane S-sulfimides, which are isoelectronic analogues of 4-silathiane S-oxides (Figure 7), also have very low activation barriers from 4.4 to 4.7 kcal/mol [52]. For N-phenylsulfonyl-4-silathiane S-sulfimide (Figure 7) the conformational equilibrium is almost degenerate (1:1), whereas in N-triflyl-4-silathiane S-sulfimide the equilibrium is shifted to the axial conformer (~55:45) due to electronegative CF_3_ group [52]. It should be mentioned, that in addition to the *ax* and *eq* conformers of the sulfimide motif, two rotamers, namely, with the “inward” or “outward” CF_3_ group may exist [53].

It also deserves mentioning that the value of ***A***_Me_ in 3-methyl-3-silathiane is 0.35 kcal/mol [35], that is, larger than in the absence of sulfur atom in the ring (0.23 kcal/mol, Table 1). A detailed analysis allowed us to conclude that the conformational preferences are governed not only by the ***A*** values of the substituents at the silicon atom, but the donor-acceptor interactions between the orbitals of the vicinal bonds play the decisive role; the degree of these interactions was evaluated by the use of the NBO analysis [35].

## 5. Azasilacyclohexanes (azasilinanes) and Related Compounds

**Figure 11:**
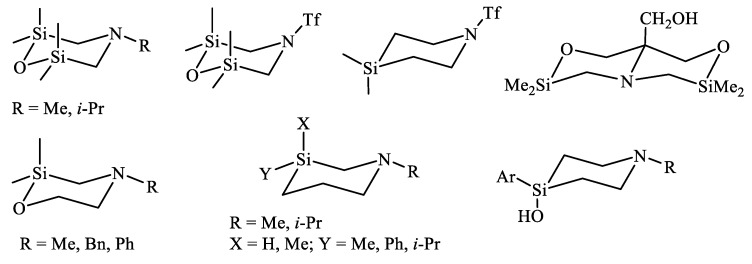
Structurally and conformationally studied azasilacyclohexanes (azasilinanes).

The structures, for which the conformational analysis was done, are given in Figure 11. The results of this analysis should be compared to those for the structures in Figure 7, to determine the effect of the nature of the heteroatom in the ring, and to the silicon-free analogues, like morpholines and piperidines.

The barriers to ring inversion in 4-alkyl-2,2,6,6-tetramethyl-1,4,2,6-oxaazadisilinanes amount to 8.1 ± 0.4 kcal/mol [54], which is less than 11.1 kcal/mol in N-methylmorpholine without α-methyl groups to oxygen [55,56]. Again, this is due to the longer Si–C than C–C bonds and less folded SiOSi part of the molecule.

**Figure 12:**
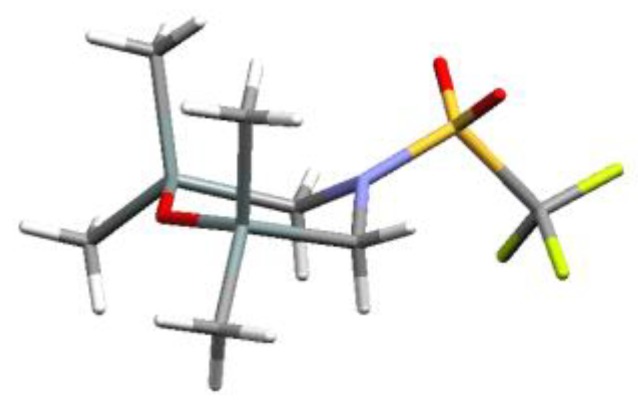
‘Inward’ rotamer of 2,2,6,6-tetramethyl-4-triflyl-1,4,2,6-oxazadisilinane.

The strongly electron acceptor triflyl group at the nitrogen atom makes it planar, but still two rotamers with the ‘inward’ or ‘outward’ CF_3_ group differing in energy by 1.1 kcal/mol in favor of the latter may exist. According to the X-ray analysis, in the crystal the compound exists as the single ‘outward’ rotamer [57] (Figure 12).

For N-triflyl-4-silapiperidine, the MP2 calculated Δ*E* is 0.6 kcal/mol in favor of the ‘outward’ rotamer, corresponding to the ratio of 95:5. The LT ^13^C-NMR spectroscopy showed the presence of two conformers in the ratio 98:2, which excellently coincides with calculations [57].

The experimental barriers to ring inversion in the N-triflyl compounds in Figure 11 are 12.9 ± 0.2 kcal/mol. An intriguing question is why the barriers in the molecules with almost planar nitrogen are higher than in non-planar N-alkyl derivatives? The answer is that these barriers refer to the interconversion between the *outward* and *inward* rotamers, rather than to the ring inversion. Since the total reaction rate cannot be larger than the rate of its limiting step, the overall barrier must be equal to that for the slowest step of the process. The conversion of rotamers of N-triflyl heterocycles is characterized by the barriers of 12–14 kcal/mol [58,59,60].

The crystal structure of a series of N-arylated N-hydroxy-1,3-azasilinanes (4-aryl-4-hydroxy-4-silapiperidines) was determined by Tacke et al. who reached the conclusion that the energy difference between the isomers of silapiperidines is notably smaller than in the corresponding piperidines [61,62,63,64,65]. In 1,4,2-oxaazasilinanes with different substituents at nitrogen in Figure 11, the ring inversion barriers decrease in the order 8.85 (R = Me) > 7.7 (R = Bn) > 4.8 (R = Ph) kcal/mol [66], that means, with increased conjugation of N_LP_ with R. Their quaternization by MeI allowed to prepare the corresponding salts and to investigate them by LT NMR; for the N,N-dimethyl salt, the degenerate conformational equilibrium is too fast even at 103 K, but for the Me,Bn-salt it was frozen and the barrier of interconversion of the conformers equal to 6.1 kcal/mol and the conformational ratio Me_ax_Bn_eq_:Me_eq_Bn_ax_ = 60:40 were found [66].

In solution, the simplest representative of 1,3-dimethyl-1,3-azasilinanes (X = H, Y = R = Me) gives an equilibrium mixture SiMe_ax_:SiMe_eq_ = 1:2 (33:67 or 30:70 from ^1^H- or ^13^C- LT NMR) [65]. However, in gas phase the axial conformer predominates and the ratio inverts to 2:1 [66]. This corresponds to the Gibbs free energy difference of –0.21 kcal/mol, which is lower than in 1,3-dimethylpiperidine (−1.6 kcal/mol [67]) but almost equal to that in 1-methyl-1-silacyclohexane (−0.23 kcal/mol [5]). Thus, the influence of the nitrogen atom on the barrier to ring inversion in 1,3-dimethylpiperidine (1.60 versus 1.76 kcal/mol in methylcyclohexane) is low (~10%) but measurable, while in silaheterocyclohexanes it is practically zero (0.02 kcal/mol).

1,3,3-Trimethyl-1,3-azasilinane was the first Si,N-heterocycle for which the gas phase structure was obtained [68]. The angle of folding between the C2Si3C4 plane and the N1C2C4C5 plane was found to be ~40° and the angle between the C6N1C2 and C2Si3C5C6 planes − ~60°.

For the Si-chiral 1,3-azasilinanes in Figure 11 (X = Me, Y = Ph, R = Me or *i*-Pr), the equilibrium is not degenerate, and the conformer ratio was measured as 67:33 (R = Me) [30] or 58.5:41.5 (R = *i*-Pr) [69], in both cases in favor of the Ph_eq_,Me_ax_ conformers. The ring inversion barriers were determined to be equal to 9.0 kcal/mol for R = Me [30] or 8.25 kcal/mol for R = *i*-Pr [69].

**Figure 13:**
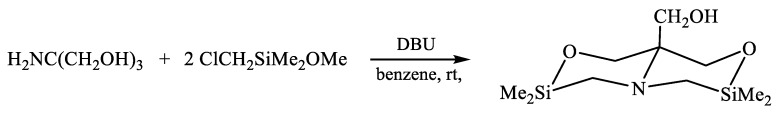
Synthesis of (3,3,7,7-tetramethylhexahydro-1*H*-[1.4.2]oxazasilino[4,5-*d*][1.4.2]oxaazasilin-9a-yl)-methanol by condensation/cyclization of 2-amino-2-(hydroxymethyl)propane-1,3-diol with ClCH_2_SiMe_2_OMe.

Among the investigated Si,N,O-heterocycles, an interesting object was the silicon analogue of quinolizidine, (3,3,7,7-tetramethylhexahydro-1*H*-[1.4.2]oxazasilino[4,5-*d*][1.4.2]oxaazasilin-9a-yl)-methanol, which was prepared by the reaction of aminoalcohol H_2_NC(CH_2_OH)_3_ with (chloromethyl)(methoxy)dimethylsilane [70] (Figure 13).

**Figure 14:**
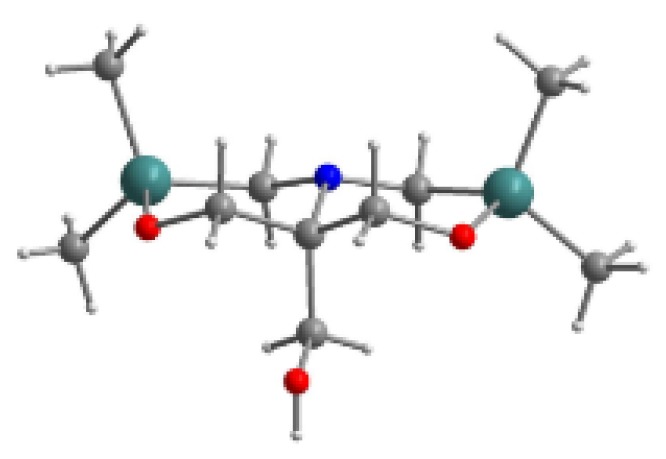
X-ray structure of 3,3,7,7-tetramethylhexahydro-1*H*-[1.4.2]oxazasilino[4,5-*d*][1.4.2]oxaaza-silin-9a-yl)methanol.

X-Ray analysis proved the *trans*,*trans*-fused structure in the crystal (Figure 14), but the molecule was conformationally flexible and the barrier for interconversion of the conformers was only 5.8 kcal/mol [70].

**Figure 15:**
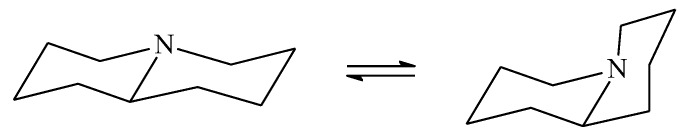
Conformational flexibility of 3,3,7,7-tetramethylhexahydro-1*H*-[1.4.2]oxazasilino[4,5-*d*][1.4.2]oxaazasilin-9a-yl)methanol due to nitrogen pyramid inversion.

Such a flexibility might be suggestive of the *cis*-fused structure of the two rings in solution, because in decalin the ring inversion is possible only in the *cis*-isomer, whereas the structure of the *trans* isomer is rigid. This seeming discrepancy can be rationalized by the fact that in N-fused compounds, such as quinolizidine, inversion at nitrogen atom is possible, as shown in Figure 15.

**Figure 16:**
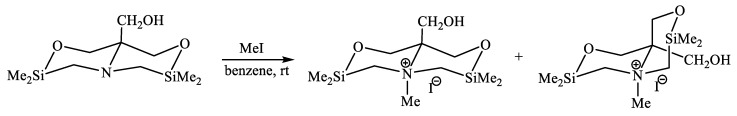
Isomeric salts formed upon quaternization of 3,3,7,7-tetramethylhexahydro-1*H*-[1.4.2]oxazasilino[4,5-*d*][1.4.2]oxaazasilin-9a-yl)methanol.

Quaternization of the nitrogen atom prevents N-inversion and the isomeric ammonium salts do not suffer interconversion. When treated with methyl iodide, 9a-R-substituted quinolizidines almpost quantitatively give the corresponding salts (Figure 16) existing as the *cis*/*trans* isomeric mixtures, in which the molar fraction of the *trans* isomer decreases in the following order: H > CN > CH_3_ > CH_2_OH > CH_2_NO_2_ [70].

## 6. Oxasilacyclohexanes (Silatetrahydropyrans)

**Figure 17:**
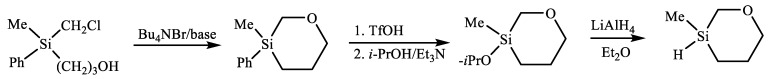
Synthesis of 3-methyl-3-silatetrahydropyran.

Some oxygen-containing silacyclohexanes with the endocyclic oxygen as the second heteroatom in the ring (3-silatetrahydropyrans) have been considered above (see Figure 4 and [32,33,34]. They include disubstituted at silicon compounds with (Me, F), (Me, OPr-*i*) and (Me, Ph) pairs of substituents. The simplest Si-chiral compound of this series, 3-methyl-3-silatetrahydropyran, was synthesized by the base-catalyzed cyclization followed by dephenylation and reduction [71] as shown in Figure 17. The problem was that it is the most volatile compound as compared to other analogues having boiling point of 62 °C at 104 mm Hg and is easily lost with the solvent during isolation.

In freons mixture solution at 103 K, ^13^C-NMR showed decoalescence of the SiMe, C4 and C5 signals and allowed to measure the conformational ratio and the ring interconversion barrier. As expected, due to small conformational energy ***A***_Si_(Me) of 0.23 kcal/mol the Me_ax_:Me_eq_ ratio was 35:65. The activation barrier was very small, 4.6 kcal/mol [71]. In gas phase the Me_ax_ conformer slightly predominates (54:46). The low energy difference between the conformers was in agreement with the theoretically calculated by most of the used DFT and MP2 methods [71].

**Scheme 1:**
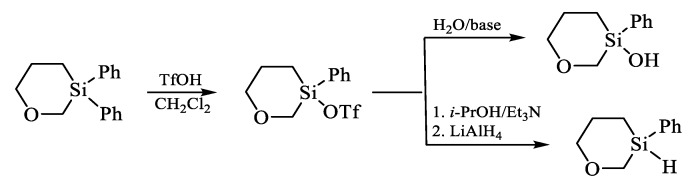
Synthesis of 3-phenyl-3-silatetrahydropyran and 3-hydroxy-3-phenyl-3-sila-tetrahydropyran.

The Si-phenyl analogue of the above compound, 3-phenyl-3-silatetrahydropyran, and the corresponding silanol, 3-hydroxy-3-phenyl-3-silatetrahydropyran, have been studied by ^13^C LT NMR and theoretical calculations. Both compounds were synthesized by partial dephenylation from 3,3-diphenyl-3-silatetrahydropyran, as shown in Scheme 1 [72].

Unfortunately, no decoalescence of the ^13^C signals was reached for 3-hydroxy-3-phenyl-3-silatetrahydropyran at the lowest temperature, so, the conformational equilibrium could not be measured experimentally. There could be because of too low coalescence temperature (<100 K) or completely one-sided conformational equilibrium. From our experience, both reasons are hardly probable, so the only feasible reason seems to be a small Δδ(^13^C) between the conformers. For 3-phenyl-3-silatetrahydropyran, however, the equilibrium was frozen and the ratio Ph_ax_:Ph_eq_ was measured as 17.1:82.9, corresponding to *K* = 4.59 and Δ*G*° = −0.31 kcal/mol [72].

## 7. Si-X-silacyclohexanes (X = Hlg, CN, OMe)

Si-Halogenated silacyclohexanes were conformationally studied by I. Arnason et al. (Hlg = F [10], Cl, Br, I [73]). The results are compiled in Table 4. For comparison, the recently published data for 1-cyano- [74] and 1-methoxy-1-silacyclohexane [75] as well as disubstituted silacyclohexanes with the phenyl group and Hlg = F, Cl [76] or other electronegative groups at silicon are included. Note, that 1-bromo-1-phenylsilacyclohexane has also been synthesized [77] but, because of low stability, its conformational analysis could not be performed.

As follows from Table 4, for R = H, all electronegative substituents prefer axial locations in the gas phase, the prevalence of the *ax* conformer varying from 54 to 84%. For X = Hlg, the same is true in solution; for X = OMe, no decoalescence was observed in the NMR spectra, but low-temperature Raman spectroscopy also suggested slight predominance of the *ax* conformer. An interesting exception is strong predominance of CN_eq_ in solution, that is, practically the same as in its carbon predecessor, cyanocyclohexane C_5_H_11_CN, exists predominantly (37:63%) as proved by GED in gas and by dynamic NMR in solution [78]. The dramatically different conformational behavior of 1-cyano-1-silacyclohexane in solution was assigned by the authors to a strong solvation effect. The conclusion was made based on the NBO analysis that the equatorial conformer is favored by the conjugation and steric effects rather than electrostatic effect. However, steric effect of the cyano group is negligible, so it was reasonably concluded that studying of Si-CN containing compounds “remains a challenging mystery” and deserve further investigation.

For R = Ph, predominance of conformer Ph_eq_Cl_ax_ in all phases and of Ph_eq_F_ax_ in solution is in agreement with equatorial preference of the more bulky phenyl group and axial preference of more electronegative halogen atom. In light of this, the predominance of Ph_ax_F_ax_, though small, is puzzling. To rationalize the observed conformational ratios, the energy partitioning analysis was employed [76].

## 8. Solution vs. Gas Conformational Preferences in Miscellaneous Silacyclohexanes

As stated above, silacyclohexanes may show inversion of conformational preferences in going from gas to solution, which is not observed for their carbon predecessors. One of the first examples was 1,3-dimethyl-1,3-azasilinane existing predominantly as SiMe_eq_ conformer (2:1) in solution but as SiMe_ax_ (with the same predominance) in gas phase [66]. Replacement of nitrogen atom in the ring by oxygen (in 3-methyl-3-silatetrahydropyran [71]) or by sulfur (in 3-methyl-3-silathiane [35,79]) does change this trend – in all these heterocyclohexanes the conformational preference is inverted in going from gas to solution. Note, that in the absence of heteroatom, the Me_eq_ conformer predominates in both aggregate states [5]. Similar conformational behavior was found for 1-phenylsilacyclohexane, for which strong preference of Ph_eq_ conformer was found in solution (78%) [6] and somewhat less predominance was measured in gas phase (62 ± 10%) [80].

**Figure 18:**
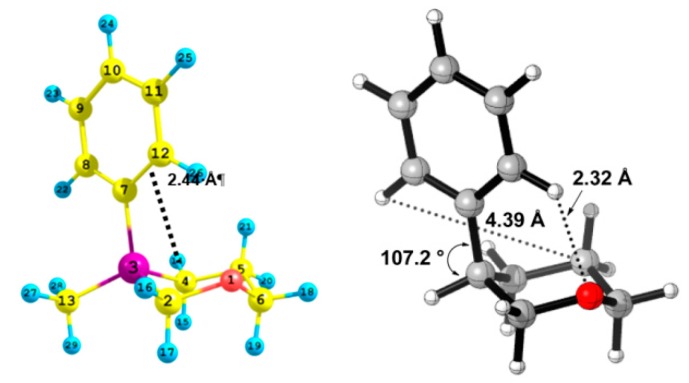
Structure of 3-methyl-3-phenyl-3-silatetrahydropyran [32] (**left**) and 3-phenyltetrahydropyran [82] (**right**).

1-Methyl-1-phenyl-1-silacyclohexane containing both methyl and phenyl substituents at silicon was studied first by low-temperature NMR in solution and showed the Ph_eq_Me_ax_:Ph_ax_Me_eq_ ratio of 63:37% at 103 K [6]. In gas phase, the ratio inverted being (42 ± 15):(58 ± 15)%. Therefore, the analysis of conformational preferences in this and related compounds allows to conclude that in gas phase at room temperature, the methyl group in the Me/Ph geminally substituted compounds shifts the conformational equilibrium towards the Ph_ax_ conformers, from Ph_eq_:Ph_ax_ = 100:0 in phenylcyclohexane [7] to 28:72 in 1-methyl-1-phenylcyclohexane [19] and from 62:38 in 1-phenylsilacyclohexane [80] to 42:58 in 1-methyl-1-phenyl-1-silacyclohexane [81]. Introduction of oxygen atom into the latter molecule gives 3-methyl-3-phenyl-3-silatetrahydropyran and increases the Ph_ax_/Ph_eq_ ratio in the gas phase from 1.38 to 1.63, which was assigned to H*_ortho_*···O interaction in the Ph_ax_ conformer, similar to that in 3-phenyltetrahydropyran [82] (Figure 18).

This assumption is in agreement with the change of the conformer ratio of 1-methyl-1-phenyl-1-silacyclohexane in solution in favor of the Ph_eq_ conformer because the oxygen lone electron pair in solution is involved in H-bonding with more acidic protons of the solvent (CHCl_3_, CH_2_Cl_2_, freons); weak specific interaction H*_ortho_*···O does not play any role and the prevalence of the Ph_eq_ conformer is determined by somewhat larger conformational energy ***A*** of Ph relative to Me.

Our recent studies [72], [83] on 1-hydroxy-1-phenyl- and 1-methoxy-1-phenylsilacyclohexane, synthesized as described in [84], allow one to compare the gas vs. solution conformational preference of these compounds. Unfortunately, no decoalescence of the ^13^C signals could be reached for 1-hydroxy-1-phenylsilacyclohexane in solution, but for 1-methoxy-1-phenylsilacyclohexane a significant predominance of the Ph_eq_ conformer (Ph_ax_:Ph_eq_ = 31.2:68.8) was measured at 103 K [72]. In gas phase, close to equimolar ratio (~50:50) of the two conformers for 1-hydroxy-1-phenylsilacyclohexane and a strong predominance of the Ph_ax_ conformer for 1-methoxy-1-phenylsilacyclohexane (Ph_ax_:Ph_ax_ = 70:30) were measured by GED [83]. While the predominance of Ph_eq_ in solution is anticipated being in compliance with all rules governing the conformational preferences, the gas phase measurements are unexpected and extremely surprising. Both axial preference of more electronegative OH or OMe group and steric effects are in favor of the Ph_eq_ conformer in both compounds. However, if to consider the relative polarities of the C-Ph and C-O bonds by comparing charge differences Δ*q* = *q*Si − *q*C_ipso_, it turns out that ΔΔ*q* = Δ*q*(Ph_ax_) − Δ*q*(Ph_eq_) only slightly (<0.01 *e*) varies in all used methods [83]. In contrast, the polarity of the Si–O bonds calculated as Δ*q* = *q*Si − *q*O is more sensitive to the location of the OH or OMe group, and the value of ΔΔ*q* reaches >0.07 *e*. A general trend is that in both compounds the Si-O bond is more polarized in the Ph_eq_ than in the Ph_ax_ conformer. Also, in 1-methoxy-1-phenylsilacyclohexane, the Si-O bond is more polarized than in the corresponding conformers of 1-hydroxy-1-phenylsilacyclohexane. To resolve the contradiction stemming from the axial preference for more electronegative OH or OMe group, we assumed that steric factors outweigh small difference in the electronic effects in the molecules under investigation [83]. Sterically, the OMe group creates more hindrances than OH group, as is proved by the larger barriers to rotation in 1-methoxy-1-phenylsilacyclohexane (2.3 and 1.6 kcal/mol) than in 1-hydroxy-1-phenylsilacyclohexane (1.5 and 0.3kcal/mol). This may explain the observed Ph_ax_ predominance in the former (70:30) over that in the latter case (1:1).

## 9. Miscellaneous Silacyclohexanes and Related Compounds

**Scheme 2:**
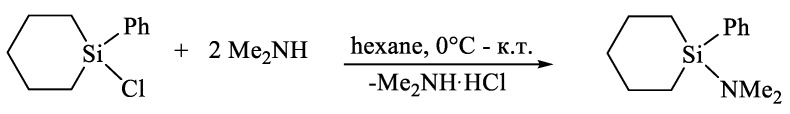
Synthesis of 1-dimethylamino-1-phenylsilacyclohexane.

An interesting type of molecular motion was found in 1-dimethylamino-1-phenyl-silacyclohexane possessing two bulky groups, Ph and Me_2_N, at silicon. The compound was synthesized in 56% yield as shown in Scheme 2. The compound is rather unstable and is gradually hydrolyzed with time to siloxane with the rupture of the Si-N bond [84].

The conformational equilibrium of 1-(Me_2_N)-1-Ph-1-silacyclohexane was studied by GED, ^13^C LT NMR and theoretical calculations [85]. The prevalence of Ph_ax_ conformer in gas phase (Ph_eq_:Ph_ax_ = 20:80%) is close to that estimated theoretically. In contrast, in solution low temperature ^13^C-NMR spectroscopy showed the predominance of the Ph_eq_ conformer, Ph_eq_:Ph_ax_ = 77:23. However, the validity of this conclusion depends on whether the assignment of the signal is correct or not. It should be said, that the predominance of Ph_ax_ conformer in the gas phase is in agreement with MP2 but not with DFT calculations. In solution, the assignment of signals in the ^13^C-LT NMR spectra is not unequivocal, either. By comparing with the spectra of 1-Ph and 1-Ph,1-X-silacyclohexanes (X = Me, OMe, F, Cl) after decoalescence [6,72,76], all experimental and theoretically calculated chemical shifts, except those for C-1,5 signal, argue for *1-Ph_eq_N_ax_ to be the preferred conformer of 1-(dimethylamino)-1-phenylsilacyclohexane*.

**Figure 19:**
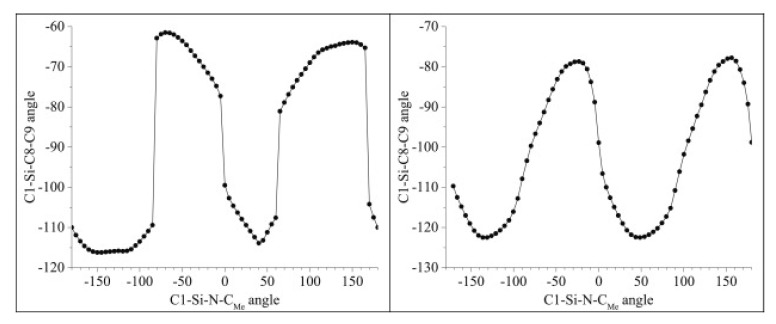
Correlation between dihedral angles C1-Si-C_ipso_-C_ortho_ and C4-Si-N-Me in the Ph_ax_ (**left**) and Ph_eq_ conformers (**right**) of 1-(dimethylamino)-1-phenylsilacyclohexane.

The potential energy surface profile for the Ph and NMe_2_ group rotation about the Si-C_Ph_ and Si-N bonds showed the presence of several conformers. In all of them, because of steric repulsion between the the *ortho*-protons and methyl hydrogen atoms, the NMe_2_ group rotation induces rotation of the Ph group, and *vice versa*. In Figure 19, the corresponding correlation plots are shown for the Ph_ax_ and Ph_eq_ conformers. As is clearly seen, rotation of the two groups is strongly correlated, which is typical for gear motion in molecular motors. ‘Vertical’ lines in the left picture appear because for two scanned angles of the NMe_2_ group differing by 5° the geometry optimization of the axial Ph group leads the system to two different local minima, causing a ‘jump’ of energy [85].

**Figure 20:**
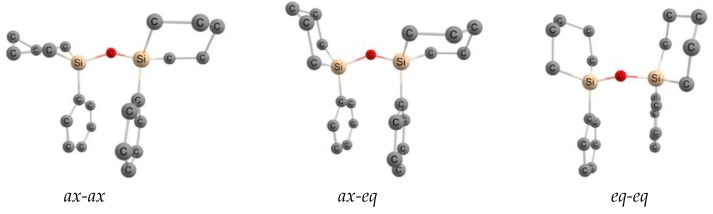
Optimized geometry (M062X/6-311G**) of possible conformers of (1,1′-phenyl-1,1′-silacyclohex-1-yl)-disiloxane. ‘*ax*’ and ‘*eq*’ refer to the position of the phenyl group.

Due to the aforementioned subordinate role of steric effects in silacyclohexanes, Ph_eq_ conformers predominate in nearly all studied 1-phenylsila(hetero)cyclohexanes [6,71]. The only exception was our recent study revealing the predominance of Ph_ax_ in 1-hydroxy-1-phenylsilacyclohexane [72]. Despite a progress in the conformational analysis of the sila(hetero)cyclohexanes having the Ph group at silicon, the question of the possibility of their existence as Ph_ax_ conformers has remained open. To fill this gap, we investigated (1,1′-phenyl-1,1′-silacyclohex-1-yl)-disiloxane. The target compound was synthesized by hydrolysis of 1-chloro-1-phenylsilacyclohexane and its ^13^C-LT NMR spectra were registered [86]. Possible conformers are shown in Figure 20.

Theoretical conformational analysis showed a slight energetic preference of the Ph_ax_ conformers (Ph_ax_,Ph_ax_ > Ph_ax_,Ph_eq_ > Ph_eq_,Ph_eq_) in spite of higher ***A*** values for the bulkier phenyl group. This was explained by a larger energy gain from shortening of the Si–O bonds in the Ph_ax_ conformers, which outweighed steric destabilization. Unfortunately, low-temperature ^1^H or ^13^C NMR studies showed the conversion of the conformers to be still fast on the NMR timescale at 100 K, so, further quantification could not be accomplished.

**Figure 21:**
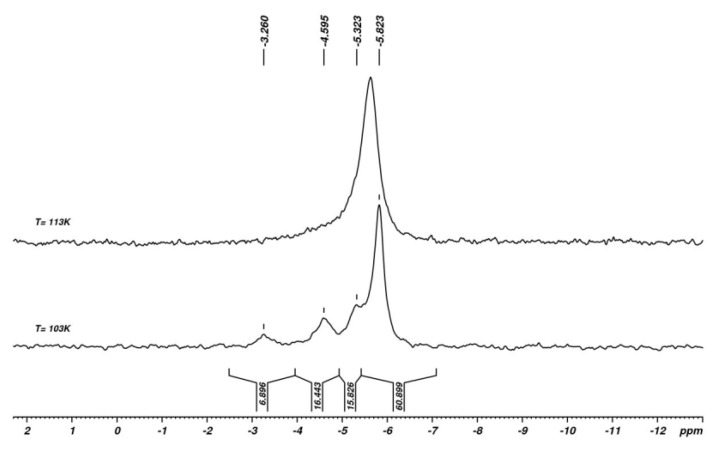
^29^Si-NMR spectra of (1,1′-phenyl-1,1′-silacyclohex-1-yl)disiloxane at 113 K (above) and at 103 K (below).

The answer to the question of conformational equilibrium of the studied siloxane was obtained from the ^29^Si-LT NMR spectra, which contained the signals of all three conformers [87]. This allowed to determine the ratio of the conformers and to assign them in accordance with the relative stability using the theoretically calculated ^29^Si chemical shifts at the GIAO/B3LYP/6-311++G(d,p)//B3LYP/6-311++G(d,p) level. The calculated shifts are reliable because (i) they lie in the interval of 2.3 ppm, which is well consistent with the experiment (2.6 ppm); (ii) the highest signal in the spectrum in Figure 21 is the most upfield one; the calculated signal for the most stable and, hence, most abundant *ax*–*ax* conformer also lies the most high field; (iii) the signal of lowest intensity is the most downfield one (Figure 21) and corresponds to the least stable *eq*–*eq* conformer; (iv) two signals of equal intensity appear between them and, judged from the relative energies of conformers in Figure 19, belong to the *ax*–*eq* conformer.

The ring inversion barrier Δ*G*^#^ measured from the temperature of decoalescence *T*_c_ and the value of Δδ of the *ax*–*ax* and *eq*–*eq* conformers was found to be one of the lowest ever measured for six-membered rings, 4.8 kcal/mol [87].

It deserves to be mentioned that the spectrum in Figure 21 represents the first example of application of the low temperature ^29^Si-NMR spectroscopy in conformational analysis [87] (cf. also Figure 6 and the text above).

**Scheme 3:**
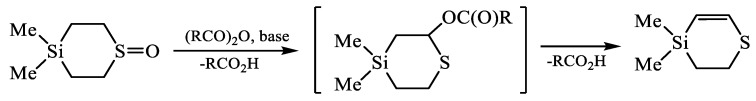
Pummerer rearrangement of 4,4-dimethyl-1,4-thiasilinane S-oxide

Interesting analogues of sila(hetero)cyclohexanes are sila(hetero)cyclohexenes. The first sulfur-containing silacyclohexene, 4,4-dimethyl-3,4-dihydro-2*H*-1,4-thiasiline, was obtained as shown in Scheme 3, in 66% yield by the rearrangement of 4,4-dimethyl-1,4-thiasilinane 1-oxide, using trifluoroacetic anhydride (R = CF_3_) [88].

**Figure 22:**
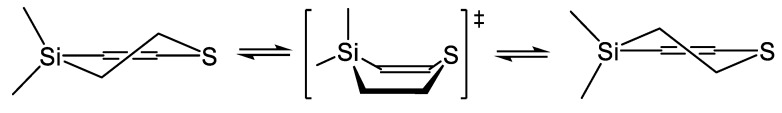
Conformational equilibrium of 4,4-dimethyl-3,4-dihydro-2*H*-1,4-thiasiline

3,4-Dihydro-2*H*-1,4-thiasilines are interesting heterocycles for conformational analysis, but till our studies there were no information on the conformational preferences even of their monoheteroatomic predecessors—silacyclohexenes or 3,4-dihydro-2*H*-thiopyrans—not to mention thiasilacyclohexenes. Moreover, due to different chains linking the silicon and sulfur atoms (Figure 22), the molecule is chiral. However, the conformational equilibrium of the synthesized 4,4-dimethyl-3,4-dihydro-2*H*-1,4-thiasiline is fast on the NMR timescale even at 103 K, assumingly, due to higher flexibility, by analogy with very flexible cyclohexene ring (ΔG^≠^ = 5.37 kcal/mol, [89]) as compared to the cyclohexane ring (ΔG^≠^ = 10.3 kcal/mol, [90]). So, we had to limit our study to theoretical computational analysis [91].

Calculations, however, did not prove a higher flexibility of the ring in Figure 22**.** Averaged barrier to the ring interconversion calculated by various methods was 5.7 kcal/mol, that coincides with that calculated by the most precise method MP2/6-311G**//MP2/cc-pVTZ [91].

**Scheme 4:**
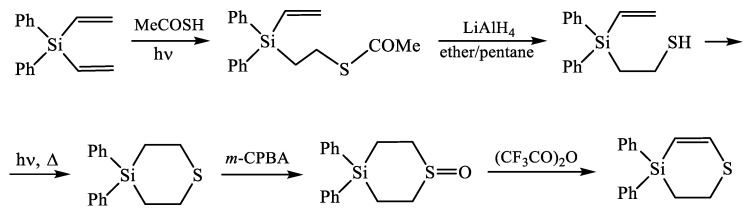
Synthetic sequence for preparation of 4,4-diphenyl-3,4-dihydro-2*H*-1,4-thiasiline.

Presuming that the phenyl groups to silicon would stabilize the compound and increase the barrier of interconversion we have synthesized 4,4-diphenyl-3,4-dihydro-2*H*-1,4-thiasiline by the same procedure as was used for the 4,4-dimethyl containing analogue [88]. The synthesis, however, was not a simple replica since some transformations were different and required a search for special reaction conditions. Full synthetic scheme, starting from diphenyl(divinyl)silane, is shown in Scheme 4.

Note, that the synthesized 3,4-dihydro-2*H*-1,4-thiasilines are the first silicon-containing cyclic vinyl sulfides. Although the target compound, indeed, turned out to be more stable chemically than its dimethylated analogue, still, no decoalescence of the signals in the NMR spectra was reached on cooling.

**Scheme 5:**
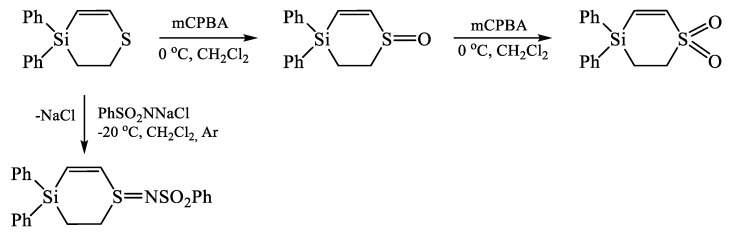
Synthesis of S-functional derivatives of 4,4-diphenyl-3,4-dihydro-2*H*-1,4-thiasiline.

**Figure 23:**
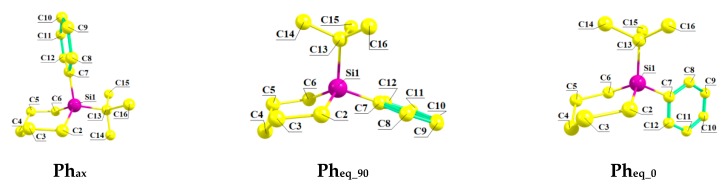
Total synthesis of 4,4-diphenyl-3,4-dihydro-2*H*-1,4-thiasiline.

The higher chemical stability and the presence of sulfur atom in the molecule prompted us to synthesize S-functionalized derivatives of the final compound in Figure 23 [92]. Another reason was very few data available even on linear S-functional derivatives of β-silylated vinyl sulfides, to say nothing of cyclic ones. The reaction of oxidation with *m*-chloroperbenzoic acid (*m*-CPBA) gives the corresponding sulfoxide in 52% isolated yield. Further oxidation to sulfone was performed using a larger excess of the oxidant in up to 60% yield (Scheme 5). We have also attempted to prepare the corresponding sulfonimide by the reaction with chloramine B. The formation of the target product was proved by the appearance of two new doublets of the SiCH= and SCH= protons, different from those in other compounds in Scheme 5, as well as by the presence of two multiplets of diastereotopic SiCH_2_ protons, as in the case of the related sulfoxide. Since sulfonimides are the least stable among the S-functional derivatives, we failed to isolate the product in the analytically pure form, because the reaction is followed by generation of a large amount of nonidentified polysiloxanes. Nevertheless, the spectral data unequivocally prove its formation.

Much lower stability of 4,4-dimethyl-3,4-dihydro-2*H*-1,4-thiasiline is characteristic also for its S-functional derivatives. During oxidation with *m*-CPBA, the compound, usually containing >10% of siloxane, gives a mixture of the sulfoxide, sulfone, and open-chain siloxanes containing the sulfoxide or sulfonyl groups [93].

Finally, the last investigated conformationally silacyclohexane was 1-*t*-butyl-1-phenyl-1-silacyclohexane, containing two voluminous substituents at silicon (our unpublished results). Both Ph and *t*-Bu groups are anancomeric in cyclohexanes, but in silacyclohexanes, only the *t*-Bu group has the ***A*** value large enough to be fully located in the equatorial position (Table 1). The synthesis of the target compound was challenging and each stage had to be optimized. After cyclization of PhSiCl_3_ with the di-magnesium derivative of 1,5-dibromopentane, BrMg(CH_2_)_5_MgBr, and subsequent treatment of the formed 1-chloro-1-phenyl-1-silacyclohexane with *t*-BuLi in pentane, the target product was obtained in 43% yield.

The conformational analysis was performed experimentally by GED and theoretically using DFT and MP2 calculations in gas phase. Three conformers were analyzed, as shown in Figure 23.

From the quantum chemical calculations, the Ph_ax_ and Ph_eq_90_ conformations are most stable, the Δ*G* value of the Ph_eq_90_ conformer being higher by 1.20–1.91 kcal/mol (cf. with the difference ***A***_t-Bu_–***A***_Ph_ of 1.05 kcal/mol in Table 1). The calculated molar fraction of the Ph_ax_ conformer varies within 88–96%, being in nice agreement with the GED experimentally measured ratio Ph_ax_:Ph_eq_90_ = 92(7):8(7)%. It can be concluded, that in the series of 1-Ph-1-X-silacyclohexanes for X = H, CH_3_, *t*-Bu, the contribution of the Ph_ax_ conformer increases from 38(10) to 58(15) and 92(8)% with increasing the X group size.

To summarize, the specific features of the structure and conformational properties of various sila(hetero)cyclohexanes and some of their derivatives are analyzed in comparison to the carbon predecessors and with an accent on the recent studies in the field. In brief, they are: (i) the determining role of electrostatic as well as stereoelectronic effects due to the presence of a highly electropositive silicon atom and strongly polarized Si-X bonds; (ii) subordinate role of steric effects due to large covalent radius of Si; (iii) much lower conformational energies of the substituents at Si; (iv) much lower barriers to ring interconversion due to substantially planarized Si-containing fragment of the ring; (v) strong dependence on the aggregate state, in many cases resulting in the inversion of conformational preferences in going from gas to the solution.

The performed analysis can be useful for studying other heterocycles containing either ‘tetrel’ elements (Ge, Sn, Pb) or the silicon atom neighbors (P, As, S, Se, etc.).

## Figures and Tables

**Table 1:** Conformational energies ***A*** (kcal/mol) for different substituents at C and Si atoms.

X	*A*(X)_C_	*A*(X)_Si_
F	0.36	−0.28 [10]
Cl	0.53	−0.43 [11]
Br	0.48	−0.82 [12]
I	0.49	−0.59 [13]
OH	0.6–1.0	0.03 [14]
SiH_3_	0.33 ^a^	0.05 [15]
Me	1.76	0.23 [5]
CF_3_	2.50	0.40 [9]
Ph	2.87	0.25 [6]
*t*-Bu	4.7	1.30 [16]

^a^ Calculated from *eq*:*ax* = 90:10 at 75 C [17].

**Table 2:**
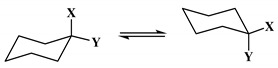
Nonadditivity of conformational effects in cyclohexanes.

X	Y	Δ*G*°_ax–eq_	Δ*G*°_add_	ΔΔ*G*°	Ref.
Me	F	0.86	1.60	−0.74	[18]
Me	CF_3_	0.53	1.31	−0.78	[18]
Me	Ph	0.32	−1.11	1.43	[19,20]
Me	NMe_2_	−0.4	0.21	−0.61	[21]
Ph	NMe_2_	−0.5	1.34	−1.84	[22]
Me	OH	0.31	0.73	−0.42	[23,24]
Ph	OH	0.5	1.86	−1.36	[25]

**Table 3:**
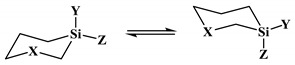
Additivity of conformational effects in silacyclohexanes and sila(hetero)cyclohexanes.

No.	X	Y	Z	Δ*G*°_ax–eq_	Δ*G*°_add_	ΔΔ*G*°	Ref.
1	CH_2_	Me	F	0.32	0.51	−0.19	[18]
2	CH_2_	Me	CF_3_	0.61	0.49	0.12	[18]
3	CH_2_	Me	Cl	0.36	0.36	0.00	[29]
4	CH_2_	Me	Ph	0.11	0.02	0.09	[6]
5	CH_2_	Ph	F	0.46	0.53	−0.07	[8]
6	CH_2_	Ph	Cl	0.53	0.68	−0.15	[8]
7	CH_2_	Ph	OR	0.20	(0.20)	0.00	[9]
8	CH_2_	Ph	*t*-Bu	1.1 ^a^	1.05	0.05	^a^
9	NR	Me	Ph	0.10–0.24	(0.02)	(−0.08–(−0.22))	[30]
10	NR	Me	OR	−0.21	(0.20)	**(−0.41)**	[31]
11	O	Me	Ph	0.16	(0.02)	(0.14)	[32]
12	O	Me	OR	0.02	(0.20)	**(−0.18)**	[33]
13	O	Me	F	0.37(gas) >1.4(solution)	0.51 0.51	0.14 **>0.9**	[34] [34]
14	S	Me	Ph	0.15	0.25	−0.10	[6]
15	S	Me	F	−0.78	−0.79	0.01	[35]

^a^ Lower limit of Ph_ax_*t*-Bu_eq_:Ph_eq_*t*-Bu_ax_ = 92(7):8(7) (GED, 337 K; our unpublished results).

**Table 4:** Conformational preferences of Si-halogenated silacyclohexanes (CH_2_)_5_Si(R,Hlg).

R	X	X*_ax_* Conformer		
		Gas	Solution	Ref.
H	F	63.0	64.2	[10]
	Cl	62.5	83.0	[73]
	Br	70.5	85.7	[73]
	I	54.0	85.7	[73]
	CN	84	35	[74]
	OMe	59	− *	[75]
Ph	F	40	76	[76]
	Cl	79	82	[76]

* No decoalescence in NMR. Slight predominance of the *ax* conformer according to Raman spectroscopy.
